# Understanding cartilage protection in OA and injury: a spectrum of possibilities

**DOI:** 10.1186/s12891-020-03363-6

**Published:** 2020-07-03

**Authors:** Anand O. Masson, Roman J. Krawetz

**Affiliations:** 1 McCaig Institute for Bone & Joint Health, University of Calgary, Calgary, AB Canada; 2 Biomedical Engineering Graduate Program, University of Calgary, Calgary, AB Canada; 3 Department Cell Biology and Anatomy, University of Calgary, Calgary, AB Canada; 4 Faculty of Medicine, University of Calgary, 3330 Hospital Drive NW, Calgary, Alberta T2N 4N1 Canada

**Keywords:** Chondroprotection, Regeneration, Cartilage, Animal models, Osteoarthritis

## Abstract

**Background:**

Osteoarthritis (OA) is a prevalent musculoskeletal disease resulting in progressive degeneration of the hyaline articular cartilage within synovial joints. Current repair treatments for OA often result in poor quality tissue that is functionally ineffective compared to the hyaline cartilage and demonstrates increased failure rates post-treatment. Complicating efforts to improve clinical outcomes, animal models used in pre-clinical research show significant heterogeneity in their regenerative and degenerative responses associated with their species, age, genetic/epigenetic traits, and context of cartilage injury or disease. These can lead to variable outcomes when testing and validating novel therapeutic approaches for OA. Furthermore, it remains unclear whether protection against OA among different model systems is driven by inhibition of cartilage degeneration, enhancement of cartilage regeneration, or any combination thereof.

**Main text:**

Understanding the mechanistic basis underlying this context-dependent duality is essential for the rational design of targeted cartilage repair and OA therapies. Here, we discuss some of the critical variables related to the cross-species paradigm of degenerative and regenerative abilities found in pre-clinical animal models, to highlight that a gradient of regenerative competence within cartilage may exist across species and even in the greater human population, and likely influences clinical outcomes.

**Conclusions:**

A more complete understanding of the endogenous regenerative potential of cartilage in a species specific context may facilitate the development of effective therapeutic approaches for cartilage injury and/or OA.

## Background

As a leading cause of disability and morbidity worldwide, osteoarthritis (OA) is a degenerative joint pathology associated with significant health and economic burden to patients and society [1, 2]. The development of OA involves a series of structural changes within the joints, and it is influenced by numerous risk factors, such as aging, genetics, and injury/trauma. Despite extensive heterogeneity observed in the onset and pathogenesis of OA, the progressive degradation of the articular cartilage appears as a unifying feature, and it remains a central focus in regenerative medicine approaches to the treatment of OA.

The articular cartilage is an intricate and remarkable tissue that provides the biomechanical properties and a low friction surface necessary for the proper function of synovial joints [3, 4]. While articular cartilage grants pain-free mobility under physiological conditions, once damaged, it presents poor innate healing capacity. Moreover, common surgical interventions aiming to improve cartilage healing, such as microfracture and autologous chondrocyte implantation (ACI), often result in a fibrocartilage patch (i.e., repair) as opposed to restoring the native hyaline cartilage (i.e., regeneration). The differences in structure and composition combined with a lack of integration with the native tissue render fibrocartilage biomechanically incompatible with the articular cartilage. These shortcomings are thought to accelerate the fibrocartilage breakdown leading to further articular cartilage injury/degeneration over time [5, 6].

Attempts have been made to circumvent this poor intrinsic regenerative ability of cartilage and protect it from further degradation after damage or disease, by promoting an environment that is chondroprotective (preventing cartilage breakdown) and/or chondro-inductive (restoring cartilage) [7]. These include inhibiting catabolic-related processes [8, 9], modulating inflammation [10], favoring chondrogenesis [11, 12], and recruiting or exogenously delivering cells of various potencies (e.g., stem cells derived from various tissue sources to chondrocytes) [13–16]. Despite many efforts, however, the development of effective disease-modifying therapies for injured cartilage and OA has yet to be realized.

**Fig. 1:**
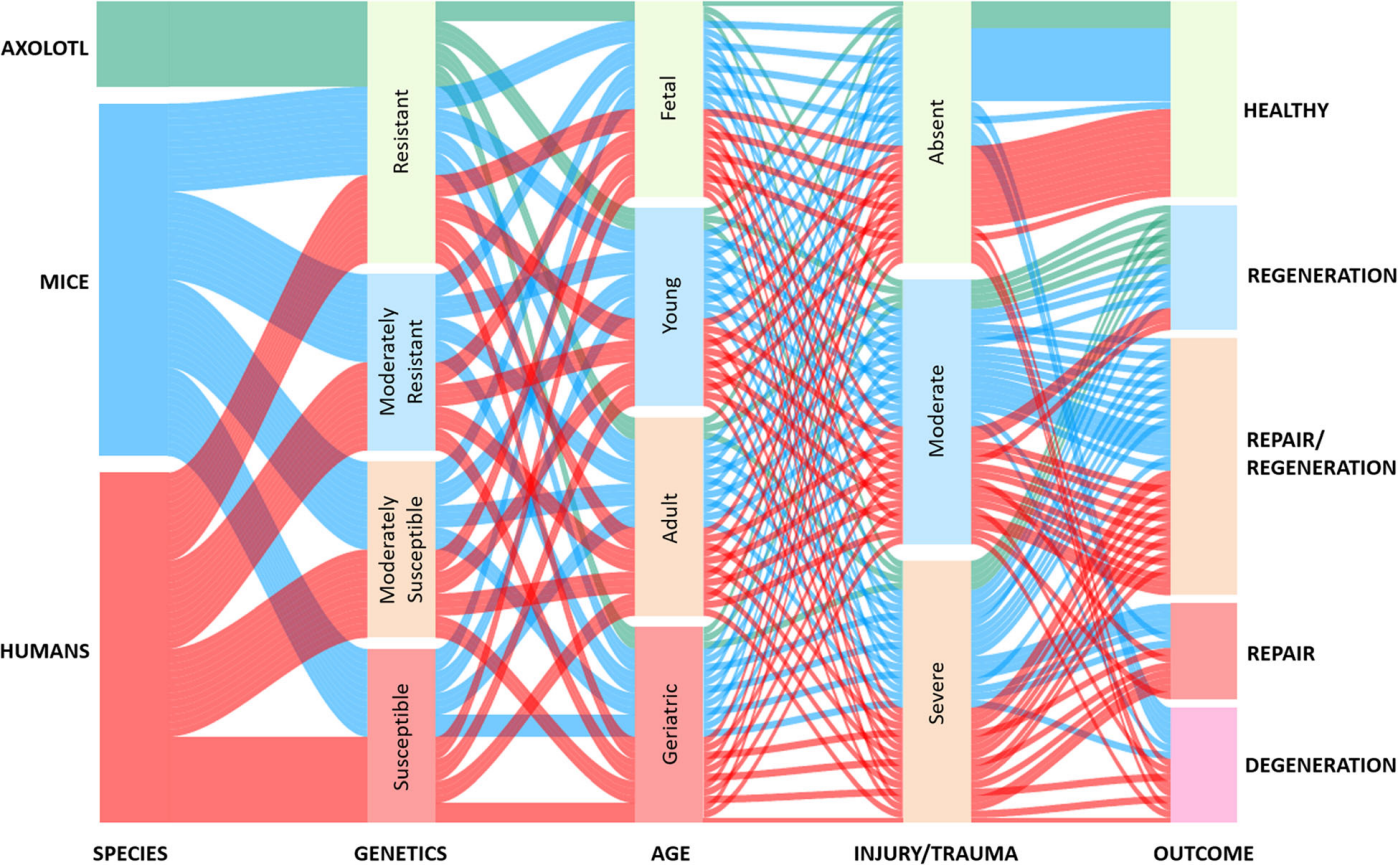
Factors influencing the regeneration and degeneration processes in cartilage. The roles of species, genetic and epigenetic traits, age, as well as the type and severity of the cartilage damage need to be considered in how this modulates the gradient of regenerative competence, homeostasis and tissue degeneration

The therapeutic potential of cell-, drug- or surgical-based interventions focused on cartilage injury and OA is commonly assessed using pre-clinical animal models. Notably, not only different species but also strains at various ages and contexts of joint damage (direct cartilage injury, post-traumatically induced or spontaneous OA) are employed. All these variables can influence the regenerative and degenerative responses, create a spectrum of outcomes (Fig. 1) and have critical implications for validating new therapeutic strategies. For instance, spontaneous and trauma-induced cartilage injuries appear to differ in terms of molecular signatures and responses to interventions [17], likely due to divergent mechanisms of disease pathogenesis. Also, age and genetic/epigenetic traits may influence the regenerative competence of animals, thus appearing as confounding factors in cartilage-related studies [17, 18].

Moreover, previous studies have reported that the rate of progression and severity of cartilage degeneration and OA-related changes in the joint differ among commonly used trauma-induced models [19, 20], such as destabilization of the medial meniscus (DMM) and anterior cruciate ligament transection (ACLT). In that sense, one might question whether the inability to protect against OA is driven by an overwhelmed endogenous repair response or the complete lack of any regenerative potential in the tissue. Another important consideration, often overlooked, is whether chondroprotective outcomes seen in OA pre-clinical studies are driven by inhibiting cartilage degeneration or enhancing cartilage regeneration, and if/how these factors may interrelate in the observed outcome. Despite the critical role that the balance between cartilage regeneration and degeneration plays in tissue maintenance and homeostasis, few studies provide a comprehensive view of how specific treatments contribute to the prevention of cartilage degeneration, in the context of cartilage regeneration (or vice versa) within a given model system [21, 22].

Therefore, in this review, we provide an overarching view of some of the key variables and their impact on cartilage tissue regeneration and degeneration. Also, we discuss how understanding the baseline of the endogenous regenerative capacity within pre-clinical models, and its modulation within a conducive environment is essential and should be integrated into the assessment of therapeutic approaches for cartilage injury and OA, in a context-specific manner.

### Main text - factors influencing regeneration and degeneration of cartilage

#### Diversity of regenerative potential across animal models

While most tissues in mammals often fail to regenerate, as opposed to more primitive organisms such as amphibians, a certain inherent capacity to respond to injury is present. Regenerative competence after distal digit tip amputation, for instance, has been reported in mice [23, 24] and similarly in the fingertip of humans [25], and shown to persist to some degree into adult life [26]. Yet, it remains unclear if fingertip regeneration like digit tip regeneration is mediated by blastema formation [24], wherein mesenchymal precursors contribute to the multi-tissue regeneration. Endogenous appendage regeneration involving cartilaginous tissues has also been identified after through-and-through ear punches in different species [27, 28], and interestingly in the antlers of deer, which are known to regenerate periodically and naturally, as well as after injury or amputation [29].

Various animal models have been employed in cartilage injury and OA-related studies, including mice, rats, guinea pigs, rabbits, dogs, and larger animals such as goats and horses [30–36]. Each animal model presents advantages and limitations in terms of their cost-benefit, suitability to mechanistic and molecular studies, and translational potential (i.e., relevance to human OA), all of which have been previously reviewed [35, 37]. However, diversity in regenerative potentials across and intra-species is also known to exist and can influence the outcome of cartilage-related studies.

Rabbits have been shown to possess robust intrinsic healing compared to humans, with previously reported wound regeneration of ear biopsy punch [27, 38] and superior healing response to full-thickness cartilage lesions [5, 39]. Caution has also been advised on the use of mice and rats given their persisting open growth plates as adults [30], which possibly enhances the natural healing of articular cartilage. However, contradictory to such belief, age-related decline in the regenerative potential of cartilage has been demonstrated within rodents, including among different strains of mice [40, 41]. Larger animals such as dogs and horses, on the other hand, seem to mimic the lack of intrinsic cartilage healing generally observed in humans, and thus are often considered as more appropriate models to evaluate the translational potential of clinical treatments for OA. Despite that, the genetic diversity within larger animals is regarded as a source of variability in cartilage repair studies [30], which possibly has wide-reaching implications on the outcomes. Therefore, unraveling the genetic/epigenetic differences that drive heterogeneity may help us understand why specific individuals are protected from OA, whereas others are more susceptible to its development.

While a comprehensive genome screening of the greater human population remains elusive, mouse studies can provide some insights into how genetic variations might be associated with OA resistance or vulnerability [34]. Murine models are powerful tools in the investigation of specific genes related to mammalian tissue regeneration, owing to the ease and sophistication of current genetic manipulations, abundant availability of recombinant inbred lines, and a broad-spectrum of cartilage regenerative potential among strains, from healers to non-healers [42], to those displaying spontaneous cartilage degeneration [43]. Gaining a better understanding of model systems which display endogenous cartilage regeneration at the molecular/genetic level, will inform us why these processes are ineffective in non-healing model systems, often leading to the development of OA.

#### Effect of genetic makeup: spectrum from endogenous regeneration to spontaneous degeneration

Overall, few mammalian model systems demonstrate robust cartilage regeneration in vivo. In mice, it has been observed in the Murphy Roths Large (MRL/MpJ) strain, whose superior ability to regenerate cartilaginous tissue was first demonstrated in the ear pinnae after through-and-through punch wound [28] and later in the knee joint following a full-thickness cartilage defect (FTCD) [44]. The Sandell group has demonstrated a strong correlation between auricular (ear) and articular (knee) cartilage regenerative abilities post-injury, as well as protection from OA [6], such that the healing phenotype is associated with a heritable component [45]. The parental strain LG/J, which shares 75% of MRL/MpJ genome, and the LGXSM-6 intercross, which shares 76% of LG/J genome, have also been found to exhibit similar regenerative abilities [6].

Subsequent studies showed that MRL/MpJ regenerative abilities extend to other tissues [46, 47], and more in-depth investigations provided insightful information regarding the mechanisms underlying its superior cartilage regeneration*.* Of note, disturbance of the cell cycle machinery including increased DNA damage and decreased levels of p21 protein, known as a critical cell cycle regulator, were identified in cells derived from MRL/MpJ mice. Later, the enhanced healing potential of through-and-through ear injuries was observed in p21 knockout (p21^−/−^) mice, suggesting the lack of p21 is at least partially responsible for the enhanced regenerative phenotype seen in MRL/MpJ mice [48]. This has been further corroborated by recent findings implicating p21 deletion in articular cartilage regeneration [49]. However, the involvement of p21 in other intricate cellular processes, such as apoptosis [50] and inflammation [51], in addition to its tight regulation, hinders our ability to elucidate the exact mechanisms associated with the healing phenotype seen after its deletion [52]. Overall, the molecular pathways controlling tissue regeneration within the abovementioned models remains unclear and of great interest in regenerative medicine.

More recently, multi-tissue regeneration has been identified in the African spiny mice (*Acomys*), including scar-free healing of auricular cartilage after ear biopsy punch [53]. Yet, no evidence of articular cartilage regeneration has been shown within this model to date. Notably, however, cell cycle regulation in progenitor cells was pinpointed as one of the key features separating regeneration after ear punches in *Acomys* from scarring in wild-type controls [27]. The same study revealed that a gradient of regenerative potential exists not only between species (healers and non-healers) but also within healer species, wherein ear pinna regeneration varied in closure rate and was likely influenced by factors such as sex and genetic variants [27]. While it is more natural to identify species that fall within the opposite ends of the healing spectrum, from fibrotic to regenerative responses, comprehending the differences that give rise to this healing continuum will greatly inform the design of targeted cartilage and OA therapies for humans.

In this context, understanding the processes leading to spontaneous OA pathogenesis and associated cartilage degeneration is just as important, since it is the most common form of OA in humans, affecting mainly elderly populations. Spontaneous cartilage degeneration has been reported in the Dunkin Hartley guinea pig (3 months old) [54], the commonly employed C57BL/6 mice with advanced age (> 17 months old) [55], and in the STR/Ort mouse (12 to 20 weeks of age) [56], which shares many similarities to the severity and progressive rate of joint deterioration that occurs in humans [57]. Age-dependent spontaneous degeneration has also been reported in larger species, such as dogs and horses [35, 37]. Despite recapitulating many of the patterns of disease progression described in human OA, spontaneous OA models present various challenges, such as longer experimental times and variable incidence and progression of OA between animals, likely owing to genetic variation. Moreover, aging brings about various changes in the molecular, cellular and functional levels both locally in the joint tissues and systemically in the body, all of which can influence the dynamics of OA pathogenesis and may diminish the tissue’s regenerative response.

#### Aging and the regenerative potential

The age of the animal is always an essential consideration, as it is generally agreed that younger animals have increased intrinsic cartilage regenerative potential compared to adults. Pre-clinical studies to date have demonstrated correlations between aging and regenerative decline within mammals [39, 40]. Yet, the biological and molecular mechanisms responsible for enhanced regenerative competence at a younger age remains poorly understood. Joutoku et al. have recently studied the involvement of chemokines, namely the CCL21/CCR7 axis, in regulating cartilage regeneration at a younger age [18]. Interestingly, juvenile mice deficient in CCR7 displayed significantly impaired cartilage healing post-injury (longitudinal full-thickness cartilage injury in the trochlear groove) compared to wild-type controls, while adult CCR7-deficient mice developed similar fibrocartilaginous tissue as controls in response to injury. Moreover, exogenous delivery of CCL21 ligand, whose transient expression had been identified at the injury site in juvenile mice, led to enhanced healing in adult rabbit after osteochondral defects [18]. Collectively, these findings suggest that this signaling pathway could be a promising target for the enhancement of adult tissue regeneration.

As for disease development, studies investigating correlations between spontaneous OA and aging have pinpointed the increase in senescent chondrocytes with age as an essential contributing factor to cartilage degeneration [58–60]. Additionally, diminished chondrocyte activity and consequently reduced turnover of ECM components [61, 62], a diminished or dysfunctional pool of stem cells [15, 63], oxidative stress [64], and differential expression of pro-inflammatory cytokines and chemokines [65] all seem to play a role in age-related OA. Some of these molecular features have also been shown in post-traumatically induced OA (PTOA) models [66]; however, there are few comprehensive studies exploring the synergistic effects of age and trauma, and how it influences response to treatment.

When comparing age paradigms in different strains of mice, it has been shown that OA severity in aged mice is greater than young mice following injury [17, 67, 68]. *Huang* et al. described age-dependent structural changes post-trauma in the articular cartilage and subchondral bone of mice, with OA features appearing earlier and being more pronounced in the aged groups [68]. Furthermore, *Loeser* et al. have previously reported that age also alters gene expressions in the whole joint, even in the absence of injury, highlighting this likely affects the tissue response after a traumatic event [67]. Not surprisingly, old versus young animals have been shown to respond differently to intervention [17, 60]. For instance, selective removal of senescent cells that arise in the knee joint after traumatic-injury at 10-week old mice was shown to protect them from OA development, decrease pain and promote a pro-chondrogenic environment [60]. In contrast, the clearance of senescent cells in aged mice (19-months old) was insufficient to overcome disease progression [60].

Similarly, Usmani et al. explored the therapeutic potential of inhibiting TGFα, a growth factor previously implicated in OA pathogenesis, within the context of spontaneous and post-traumatic OA. The latter employed a DMM surgery model to induce cartilage injury in young (10-week old), as well as aged (6-month old) mice. The authors found that TGF-α deficiency did not protect mice from the development of spontaneous OA and that its effect in trauma-induced OA is age-dependent, whereby only young mice were protected from OA progression [17]. Ultimately, the etiology of human OA is highly complex, thus thoughtful consideration must be given to the species, age, and disease model chosen, including if it is spontaneous or post-traumatically induced, and how appropriate each one is for the exploration of specific human clinical subtypes.

#### Models of OA and cartilage damage

Research focusing on cartilage degeneration typically employs animal models of spontaneous or post-traumatic OA to elucidate the mechanisms of onset and progression of the disease (Table 1). As previously discussed, models of spontaneous cartilage degeneration primarily explore the effects of genetic traits and aging on the susceptibility to OA, whereas PTOA models typically induce cartilage degeneration by surgically producing joint instability and altering its regional distribution of loads (e.g., ACLT, DMM, meniscectomy). Although such approaches have provided valuable information on related risk factors, diagnostic biomarkers, and potential therapeutic targets, different models can promote distinct yet intertwined pathways leading to cartilage degeneration, thus influencing the study outcome.

**Table 1:** Summary of most widely used osteoarthritis models and direct cartilage injury models with respect to their type/mode of action and most commonly employed species [40, 44, 79]

CARTILAGE DEGENERATION	Osteoarthritis models			Commonly used species
	Spontaneous	Aging	Naturally occurring	Guinea Pig, Mouse, Dog
		Genetic	Genetically modified	Mouse
	Induced	Chemical	Collagenase	Mouse, Rat, Rabbit
			Sodium Monoiodoacetate (MIA)	
		Diet-induced	Obesity/Metabolic syndrome	Mouse, Rat
		Post-traumatic (non-invasive)	Cyclic tibial compression	Mouse, Rabbit, Dog
			Intra-articular tibial fracture	
		Post-traumatic (invasive/surgical)	Anterior cruciate ligament transection (ACLT)	Rat, Rabbit, Dog
			Destabilization medial meniscus (DMM)	Mouse, Rat
			Meniscectomy	Mouse, Rat, Rabbit, Dog, Goat
CARTILAGE REGENERATION	Cartilage Injury models			Commonly used species
	Induced	Longitudinal full-thickness cartilage defect [40]	Osteochondral/Chondral defect - trochlear groove	Mouse, Rabbit
		Full-thickness cartilage defect (FTCD) [44]	Focal osteochondral defect - trochlear groove	Mouse, Rat, Rabbit, Dog, Horse

Haase et al. have recently compared two surgically induced PTOA models in C57BL/6 mice, specifically DMM and transection of the medial collateral ligament (MCL-MM) [20]. Mice that underwent MCL-MM demonstrated rapid and pronounced degradation of the collagen matrix component, with cartilage lesions being identified as early as 6-weeks post-surgery. By contrast, no cartilage lesions were seen for the duration of the assessment (12 weeks post-surgery) in the DMM model, which displayed slow OA progression, with proteoglycan loss over an extended period and identifiable collagen degradation by 8-weeks post-surgery. It is worth noting, however, that other studies have reported histological evidence of cartilage lesions after DMM surgery at earlier time-points [67–70] than the one observed in this study, which might own to factors such as sex and age of the animals at the time of injury [68]. That aside, even though mechanical destabilization serves as the initiating factor in both models, differences in the dynamics of molecular and structural changes, and associated progressive cartilage degeneration were distinct, which speaks to differential regulation of secondary mechanisms.

Of note, surgical models of PTOA are invasive, with the surgery itself inducing inflammation, and possibly degenerative changes in the joint environment, thereby diminishing our ability to understand the mechanisms underlying the disease phenotype. Therefore, non-invasive approaches mimicking PTOA have been developed, which involve mechanical overloading of the joint to induce cartilage lesions (Table 1). Some examples would be non-physiological cyclic compression and closed intra-articular tibial fracture (IAF) [71–74]. Regardless of the initial trigger, divergences in the mechanistic aspects of progression and severity of degenerative changes have been shown in comparative studies involving surgical and non-surgical PTOA models across species and strains [19, 73, 75–77].

Other examples of induced OA models, yet non-traumatic in nature, include chemically and diet-induced models (Table 1). Chemically induced models rely on the injection of compounds that promote damage to cartilage components, compromising its function, whether by means of inflammation or toxicity [78, 79]. Sodium monoiodoacetate (MIA) is one of the most widely used compounds as it promotes joint inflammation and chondrocyte death. While less invasive than surgically induced models, the rapid progressing degeneration induced by these compounds bares little resemblance to the pathophysiology of OA, thus they are more commonly employed for pain-related studies [37]. Given the relevance of obesity and metabolic syndrome associated risk in the development of OA [80], efforts have been directed to understanding their effects on the incidence and pathogenesis of the disease. Diet-induced OA models, most typically performed in rodents, expose the animals to high-fat or high-fat/high-sucrose diet regimens. These models have been shown to successfully induce OA-like joint degeneration [81–83] mainly driven by low-grade inflammation [80]. In line with other studies, age has been shown as a contributing factor in diet-induced OA severity [84]. It is interesting, however, that individuals from the same species have been reported to display distinct obesity phenotypes in response to diet-induced metabolic disturbance, and that their susceptibility or resistance can be associated with severity of OA-like knee damage [81, 85]. However, the impact of obesity and low-grade inflammation displayed in these models on cartilage regeneration has not yet been characterized.

While it is broadly assumed that articular cartilage has little to no intrinsic repair at a population level, what if cartilage regeneration can outplace cartilage degeneration in some proportion of the population. This hypothesis is supported by results from previous clinical studies, wherein only about 50% of human patients that undergo cartilage or joint injury develop OA over time [86, 87]. In this context, one might reasonably question whether the protection against OA in these individuals is driven by enhanced regenerative capability and/or muted degenerative response. Therefore, genetic background and the individual’s baseline of endogenous regenerative capacity should be considered and controlled for when comparing outcomes post-injury. While this is inherently difficult to test in humans directly, pre-clinical mouse studies support this hypothesis.

Despite their genetic predisposition to the development of spontaneous OA, STR/ort mice are less prone to cartilage lesion formation after joint compressive overloading than CBA mice [43], suggesting that inherent genetic risk of developing OA is not directly associated with susceptibility to trauma-induced cartilage damage. Interestingly, the “super-healer” MRL/MpJ mice are resistant to cartilage damage and show reduced severity of PTOA when compared to C57BL/6 mice, whether following surgical DMM [22] or non-surgical IAF [88]. Similar results are seen in the LG/J strain [34], whereas p21^−/−^ mice seem to be vulnerable to cartilage damage following DMM surgery [89], despite its superior regenerative potential after full-thickness cartilage injury [49].

Direct cartilage injury is commonly used to study the tissue regenerative potential by producing a partial or a FTCD, focally or longitudinally in the trochlear groove, that can reach into the subchondral bone (Table 1) [18, 44, 90]. These models help examine the effect of genetic manipulations and exogenously delivered treatments, such as cell therapy and tissue-engineered constructs, on cartilage regeneration. Owing to the nature of their targeted site of injury, cartilage defect models allow for a straightforward revaluation of progressive tissue healing, although the relative size of injury in smaller species somewhat limits the comparison to the human condition.

**Fig. 2:**
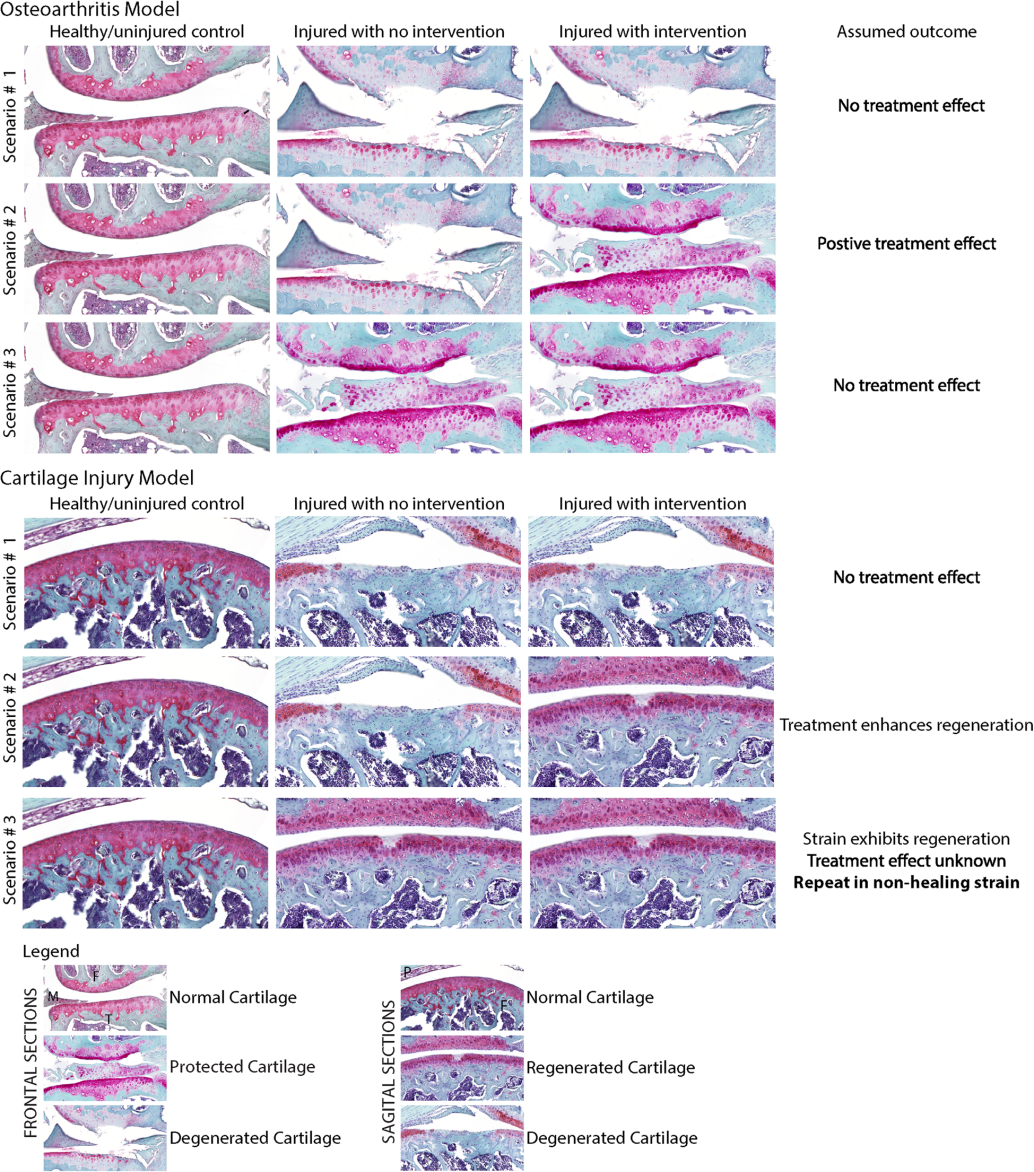
Assumed outcomes based on three different possible scenarios using an osteoarthritic model of PTOA or a FTCD cartilage injury model. In the context of indirect cartilage damage (OA model), one can analyze whether the treatment of choice is capable of attenuating or preventing OA development and cartilage degeneration (i.e., chondroprotection) when compared to untreated controls. However, the endogenous regenerative potential of the strain or species might interfere with the assumed outcome. Conversely, when using a direct cartilage injury model, one can investigate the mechanism underlying the endogenous regenerative ability, as well as to infer the efficacy of treatment of interest in enhancing cartilage regeneration

As alluded to, models of direct cartilage injury and PTOA reflect the duality of cartilage regeneration and degeneration (Fig. 2). In the context of direct cartilage injury, such as FTCD, one can investigate the mechanisms underlying the endogenous regenerative ability of a species/strain, as well as infer the efficacy of treatment of interest in enhancing cartilage regeneration. Conversely, when using an OA model, one can analyze whether the treatment of choice is capable of attenuating or preventing OA development and cartilage degeneration when compared to untreated controls.

However, to elucidate the biological mechanisms promoting a protective phenotype in the latter, it is necessary to understand whether the intervention inhibited degeneration and/or promoted regeneration of the cartilage. In which case, the causative role associated with an intrinsic healing potential and the chosen therapeutic intervention should also be investigated. Intra-articular injection of kartogenin, for instance, has been shown to protect mice against the development of OA following surgically-induced trauma [91]. Such outcome is likely due to a combinatory effect of enhanced regenerative response and chondroprotection, resulting from the modulation of endogenous stem cells and expression of chondrogenic factors, and the promotion of a conducive environment with decreased expression of catabolic enzymes, respectively [91, 92].

Recent studies investigating the involvement of chemokines and correlated inflammatory component in OA pathogenesis have linked the CCL2/CCR2 signaling axis, mainly known for its role in monocyte recruitment, to trauma-induced and age-associated OA phenotypes in humans and rodents [93–95]. Interestingly, controversy remains regarding CCL2/CCR2 contribution to cartilage degeneration. Using a murine DMM model, Miller et al. found that while depletion of CCR2 improved pain-associated outcomes, it did not protect CCR2^−/−^ mice from cartilage degeneration [96]. Expanding on these findings, Zarebska et al. reported similar outcomes due to ligand deficiency, with CCL2^−/−^ mice showing decreased pain and comparable histopathological scores to CCR2^−/−^ and wild-type controls after induced-PTOA [97]. Yet, statistical significance was reached by 20-weeks following DMM in CCL2^−/−^ mice, associated with less severe cartilage degeneration compared to the other mouse groups [97].

Likewise, Raghu et al. demonstrated that CCL2 deficiency was protective of cartilage degeneration, and promoted a significant decrease in macrophage infiltration, inflammation and expression of matrix-degrading molecules [98]. However, contradictory to previous findings, lack of CCR2 was also shown to mitigate mouse OA, whether through genetic inactivation of CCR2 or pharmacologic blockage of this receptor by a CCL2 antagonist [98]. Given such conflicting results, Jablonski and colleagues explored the association between the CCR2/CCL2 signaling axis and cartilage regeneration using the FTCD model of direct cartilage injury. Interestingly, the authors found that CCR2^−/−^, but not CCL2^−/−^ nor CCL2^−/−^CCR2^−/−^ mice display enhanced cartilage regeneration following FTCD [21]. Collectively, these results suggest that while depletion of CCL2 levels may inhibit cartilage degeneration (chondroprotective) it does not promote cartilage regeneration, whereas depletion of CCR2 is conducive of cartilage regeneration and likely associated with the controversial outcomes regarding chondroprotection post-trauma.

**Fig. 3:**
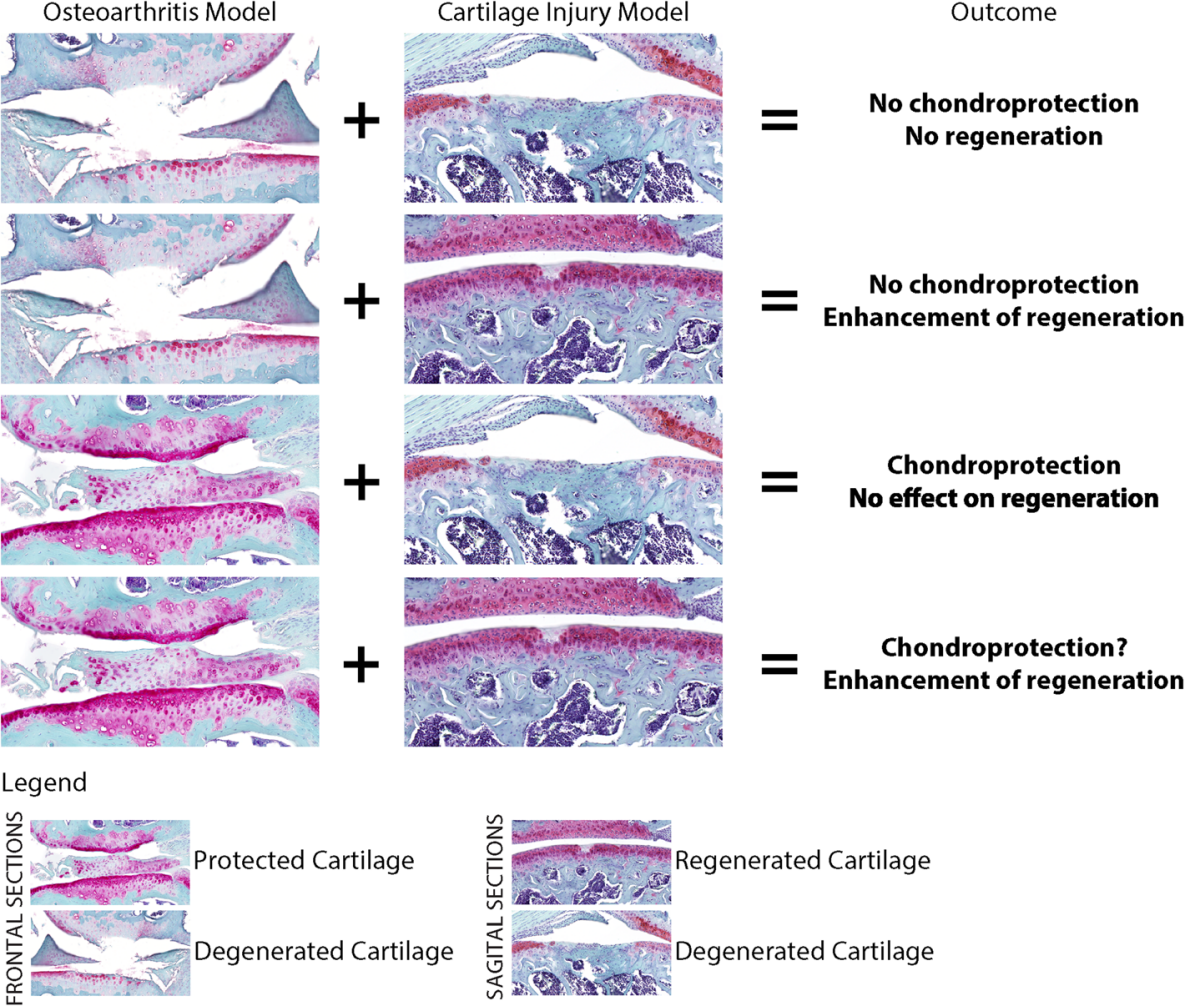
Comprehensive view of the effect of targeted treatments in inhibiting cartilage degeneration) and enhancing cartilage regeneration. The power of the combined analysis in informing the outcome of targeted therapies for OA and cartilage injury is greater than the one provided by the models isolation

Overall, studies providing a comprehensive view of how specific treatments contribute to the prevention of cartilage degeneration, as well as to the enhancement of its regeneration are scarce [21, 22]; however, the previous examples highlight the importance that a combined analysis has in informing the outcome of targeted therapies for OA and cartilage injury (Fig. 3).

## Conclusion

It is widely accepted that several risk factors can alter the progression of OA such as obesity, joint trauma, improper mechanical loading, and aging. A recent review by Mimpen and Snelling has suggested that heterogeneities in endotypes that predispose to OA onset and progression should be considered in patient selection and outcome assessment in clinical trials [99]. We suggest that the considerations highlighted in this review in terms of pre-clinical animal models may also apply to humans. There is a general belief that humans lack a regenerative response within the cartilage tissue; however, it remains unknown whether there is a gradient of regenerative competence within cartilage across the greater human population and how this might affect clinical outcomes.

We are encouraged by recent findings on the topic, suggesting that the regenerative capacity of cartilage is variable and depends on where it resides in the human body, being more robust in the ankle joints [100]. We believe it is plausible that a level of variation in endogenous regenerative response exists across patients as well, due to genetic traits or molecular mechanisms, and should also be considered in clinical studies and in the future clinical trials. In essence, a predisposition to cartilage regeneration might be present among patients that show a positive response to chondroprotective therapies. Hence, a thorough understanding of the role of treatment interventions on the dynamics of endogenous regenerative and degenerative responses may help us develop targeted and effective therapeutic approaches, wherein given a conducive environment, the regenerative stimuli can prevail among tissues and/or organs otherwise known as non-regenerative; as is the case with OA.

## Data Availability

Data sharing does not apply to this article as no datasets were generated or analyzed during the current study.

## Abbreviations

ACI: Autologous Chondrocyte Implantation
ACLT: Anterior Cruciate Ligament Transection
DMM: Destabilization of the Medial Meniscus
FTCD: Full-thickness cartilage defect
IAF: Intra-Articular Fracture
MRL: Murphy Roths Large
OA: Osteoarthritis
PTOA: Post-Traumatic Osteoarthritis
